# Thrombocytopenia in patients with melanoma receiving immune checkpoint inhibitor therapy

**DOI:** 10.1186/s40425-017-0210-0

**Published:** 2017-02-21

**Authors:** Eileen Shiuan, Kathryn E. Beckermann, Alpaslan Ozgun, Ciara Kelly, Meredith McKean, Jennifer McQuade, Mary Ann Thompson, Igor Puzanov, John P. Greer, Suthee Rapisuwon, Michael Postow, Michael A. Davies, Zeynep Eroglu, Douglas Johnson

**Affiliations:** 1 Medical Scientist Training Program, Vanderbilt University Medical Center, Nashville, TN 37232 USA; 2 Department of Cancer Biology, Vanderbilt University Medical Center, Nashville, TN 37232 USA; 3 Division of Hematology/Oncology, Vanderbilt University Medical Center, 777 PRB, 2220 Pierce Ave, Nashville, TN USA; 4 Department of Cutaneous Oncology, Moffitt Cancer Center and Research Institute, Tampa, FL 33612 USA; 5 Sarcoma Oncology Service, Department of Medicine, Memorial Sloan Kettering Cancer Center, New York, NY 10065 USA; 6 Division of Cancer Medicine, University of Texas MD Anderson Cancer Center, Houston, TX 77030 USA; 7 Melanoma Medical Oncology, University of Texas MD Anderson Cancer Center, Houston, TX 77030 USA; 8 Division of Hematology/Oncology, Lombardi Comprehensive Cancer Center, Georgetown University, Washington, DC 20057 USA; 9 Melanoma and Immunotherapeutics Service, Department of Medicine, Memorial Sloan Kettering Cancer Center, New York, NY 10065 USA; 10 Weill Cornell Medical College, Cornell University, New York, NY 10065 USA; 11 Department of Pathology, Microbiology, and Immunology, Vanderbilt University Medical Center, Nashville, TN 37232 USA; 12 Roswell Park Cancer Institute, Buffalo, NY 14263 USA

**Keywords:** Checkpoint inhibitor, PD-1, CTLA-4, Thrombocytopenia, Immune thrombocytopenic purpura, Melanoma

## Abstract

**Background:**

Immune checkpoint inhibitors, including antibodies against programmed death 1 (PD-1) and cytotoxic T-lymphocyte antigen 4 (CTLA-4), are being used with increasing frequency for the treatment of cancer. Immune-related adverse events (irAEs) including colitis, dermatitis, and pneumonitis are well described, but less frequent events are now emerging with larger numbers of patients treated. Herein we describe the incidence and spectrum of thrombocytopenia following immune checkpoint inhibitor therapy and two severe cases of idiopathic thrombocytopenic purpura (ITP).

**Case presentations:**

A 47-year-old female with recurrent BRAF mutant positive melanoma received combination anti-PD-1 and anti-CTLA-4. Two weeks later, she presented with mucosal bleeding, petechiae, and thrombocytopenia and was treated with standard therapy for ITP with steroids and intravenous immunoglobulin (IVIG). Her diagnosis was confirmed with bone marrow biopsy, and given the lack of treatment response, she was treated with rituximab. She began to have recovery and stabilization of her platelet count that ultimately allowed her to be retreated with PD-1 inhibition with no further thrombocytopenia. A second patient, a 45-year-old female with a BRAF wild-type melanoma, received anti-PD-1 monotherapy and became thrombocytopenic 43 days later. Three weeks of steroid treatment improved her platelet count, but thrombocytopenia recurred and required additional steroids. She later received anti-CTLA-4 monotherapy and developed severe ITP with intracranial hemorrhage. Her ITP resolved after treatment of prednisone, IVIG, and rituximab and discontinuation of checkpoint inhibition. In a retrospective chart review of 2360 patients with melanoma treated with checkpoint inhibitor therapy, <1% experienced thrombocytopenia following immune checkpoint inhibition, and of these, most had spontaneous resolution and did not require treatment.

**Conclusions:**

Thrombocytopenia, especially ITP, induced by immune checkpoint inhibitors appears to be an uncommon irAE that is manageable with observation in mild cases and/or standard ITP treatment algorithms. In our series, the majority of patients had mild thrombocytopenia that resolved spontaneously or responded to standard corticosteroid regimens. However, in two severe cases, IVIG and rituximab, in addition to steroids, were required. Checkpoint inhibition was resumed successfully in the first patient but rechallenge was not tolerated by the second patient.

## Background

Immune checkpoint inhibitors, including antibodies against programmed death 1 (PD-1) receptor, are quickly becoming a staple in our arsenal of anti-cancer agents. PD-1 signaling normally inactivates effector T cells when bound to its ligands PD-L1 and PD-L2; thus, inhibition of this pathway reinvigorates T cell antitumor responses [1]. Nivolumab and pembrolizumab are monoclonal antibodies that block PD-1 and have demonstrated substantial benefit in melanoma, non-small cell lung cancer, renal cell carcinoma, Hodgkin lymphoma, and numerous other cancers [2].

Although inhibitors of PD-1 provide significant therapeutic benefit, many immune-related adverse events (irAEs) have emerged with these therapies. The most common irAEs include dermatologic toxicities and thyroid dysfunction [3–5]. Other clinically significant toxicities include colitis, hypophysitis, pneumonitis, and hepatitis [4–7]. These events arise from dysregulation of self-tolerance that is normally mediated by PD-1/PD-L1 interactions [8]. Additionally, anti-PD-1 (specifically nivolumab) may be combined with ipilimumab, an antibody against cytotoxic T-lymphocyte antigen 4 (CTLA-4), which when bound to costimulatory molecules on antigen-presenting cells inactivates T cells. Ipilimumab works synergistically with anti-PD-1 agents and improves antitumor efficacy but also increases the frequency and severity of irAEs [3, 5].

Recently, cases of hematologic irAEs were reported with anti-PD-1 therapy, specifically autoimmune hemolytic anemia and immune thrombocytopenic purpura (ITP) [9–12]. Although cases of thrombocytopenia induced by either pembrolizumab or ipilimumab alone have been reported, its incidence, spectrum of severity, and development of ITP have not been established [13, 14]. Given the rapid rise of immunomodulatory therapy use in numerous cancers, there is a clear need to identify and characterize these hematologic irAEs. Here, we report two cases of severe ITP resulting from checkpoint inhibitor therapy and the largest multi-institutional case series of thrombocytopenia induced by checkpoint inhibitor therapy.

## Case presentations

**Fig. 1:**
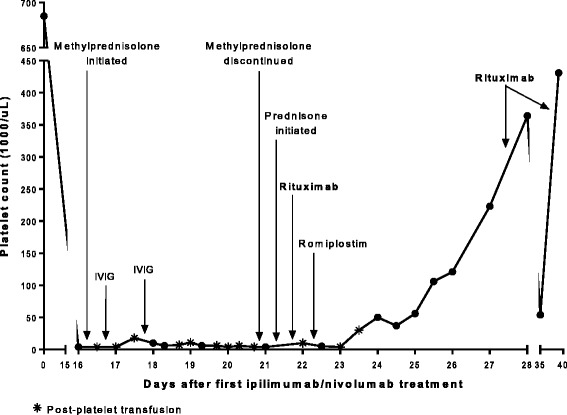
Checkpoint inhibitor-induced ITP refractory to glucocorticoids subsequently responds to second-line treatment

**Fig. 2:**
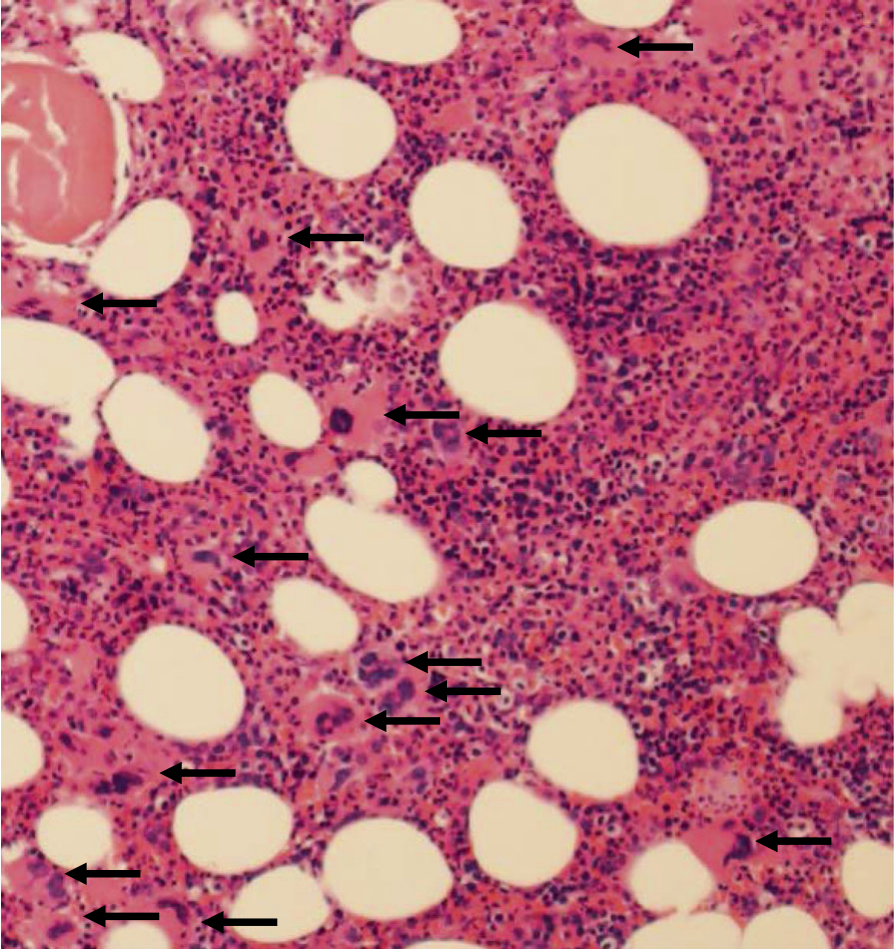
Bone marrow from patient with checkpoint inhibitor-induced ITP before rituximab treatment. H&E stained section of the bone marrow biopsy, 100 × magnification. The bone marrow is moderately hypercellular for age with trilineage hematopoiesis and increased megakaryocytes (*black arrows*) with a range of morphologies and mild clustering. These findings, coupled with the patient’s peripheral thrombocytopenia and elevated IPF, are compatible with a diagnosis of ITP

A 47-year-old patient presented in 2011 with stage IIb melanoma on her left forearm, which was removed by wide local excision with a concurrent negative sentinel lymph node biopsy. Four years later, she presented with recurrent metastatic melanoma with a *BRAF* ^V600M^ mutation. She initially responded to combination BRAF and MEK inhibition but developed progressive disease within five months. Two weeks later, she received her first infusion of combination ipilimumab and nivolumab. She developed bleeding from mucosal areas and petechiae fifteen days following her first dose of combination checkpoint inhibitor therapy with severe thrombocytopenia (PLT < 5000/uL) and an elevated immature platelet fraction of 15.4% (0.9 to 7.0% normal range). She had no history of autoimmune or coagulation disorders, and the remainder of her laboratory evaluation was unrevealing. She was presumed to have new-onset ITP and was started on methylprednisolone and intravenous immunoglobulin (IVIG) (Fig. 1). After five days of steroids and IVIG without significant improvement in her platelet count, a bone marrow biopsy revealed a hypercellular marrow with increased megakaryocytes (Fig. 2), further supporting the diagnosis of ITP. Her treatment was escalated to weekly rituximab with addition of a single dose of romiplostim, a thrombopoietin analog. Seven days after steroids and IVIG, and two days after rituximab, her platelet counts began to improve, reaching normal range (137,000 to 397,000/uL) by one week, and she subsequently did not require additional platelet transfusions (Fig. 1). Between her second and third infusion of rituximab, she transiently relapsed to a platelet count of 54,000/uL. She received a total of four doses of rituximab in accordance with standard therapy for ITP. Eight days after her last dose, she was rechallenged with nivolumab monotherapy and subsequently experienced a partial response with no relapse of her ITP.

A second patient, a 45-year-old female with a BRAF wild-type melanoma, received nivolumab as neoadjuvant therapy in a clinical trial and became thrombocytopenic (49,000/uL) 43 days later. She was asymptomatic and was treated with prednisone for three weeks, with elevation of platelet levels to baseline (307,000/uL). However, thrombocytopenia recurred with a platelet nadir of 28,000/uL, and she required an additional 12-week steroid taper before her platelets recovered. She later developed metastatic disease to the brain and underwent resection of a large frontal lesion, as well as gamma knife irradiation to several smaller lesions. Due to limited treatment options, she was administered ipilimumab monotherapy, eight months after receiving nivolumab. Within eight days, her platelet count decreased from 75,000 to 8000/uL, and two days later, she developed hemorrhage in her intracranial metastasis with no detectable platelets. Her ITP ultimately resolved after treatment with prednisone, IVIG, and rituximab and discontinuation of ipilimumab.

To assess the incidence and clinical patterns of thrombocytopenia, including potential cases of ITP, following immune checkpoint inhibition, we performed retrospective reviews of electronic medical records at Georgetown Lombardi Cancer Center, Memorial Sloan Kettering Cancer Center, Moffitt Cancer Center, MD Anderson Cancer Center, and Vanderbilt-Ingram Cancer Center. Patients with melanoma were included if they experienced thrombocytopenia following treatment with a checkpoint inhibitor that was clinically diagnosed as ITP or was not attributable to another cause. The project was approved by IRBs of respective institutions with waiver of consent. Statistical analysis was performed using R version 3.3.0.

We assessed the frequency of thrombocytopenia induced by treatment with checkpoint inhibitors across these five institutions and identified 11 cases, several presumed to be ITP based on clinical diagnostic criteria. A total of 2360 patients with melanoma receiving checkpoint inhibitor therapy were reviewed, suggesting an incidence of well under 1%. These patients were Caucasian men (58%) or women (42%) with melanoma; none had a previous diagnosis of ITP or a history of thrombocytopenia prior to initiation of treatment. Various checkpoint inhibitor regimens were represented (Table 1). The average time to onset of checkpoint inhibitor-induced thrombocytopenia was 70 days (range, 12 to 173 days), and the average platelet count was 61,000/uL (range, <5000 to 104,000/uL) with an average decrease of 70% from baseline (range, 38 to 99%). No significant differences were found among the varying checkpoint inhibitor regimens.

**Table 1:** Patients with thrombocytopenia and/or confirmed new-onset ITP following checkpoint inhibitor therapy for melanoma

	Case 1	Case 2	Case 3	Case 4	Case 5	Case 6	Case 7	Case 8	Case 9	Case 10	Case 11
Age, years/sex	52/F	80/M	55/F	44/M	67/M	45/F	53/M	48/F	36/F	56/M	69/M
Checkpoint inhibitor(s) and dosage(s)	Ipi (3 mg/kg) + nivo (1 mg/kg)	Pembro (2 mg/kg)	Ipi (3 mg/kg) + nivo (1 mg/kg)	Ipi (3 mg/kg)	Ipi (3 mg/kg) + nivo (1 mg/kg)	Nivo (3 mg/kg)	Pembro (2 mg/kg)	Pembro (2 mg/kg)	Pembro (2 mg/kg)	Nivo (3 mg/kg)	Nivo (3 mg/kg)
Best response to therapy	PR	PR	PR	PD	N/A	PD	N/A	SD	PD	SD	N/A
Time to TP onset, days	15	21	50	62	68	43	12	173	40	130	151
Other irAEs	None	Neurological	Endocrine, skin	GI	Neurological, liver	None	Neurological, liver	Skin	None	None	None
Counts at TP onset											
WBC, 10^3^/uL HCT, % PLT, 10^3^/uL	15.4 34 Less than 5	5.8 43.5 104	7 37.8 61	12.8 N/A 18	3.8 35.8 86	6 40.7 49	3.7 35.3 53	11.9 31.3 89	8.1 28.2 58	4.9 41 73	4 28.5 74
% PLT decrease from baseline	99%	38%	80%	91%	40%	84%	69%	53%	74%	N/A	75%
Signs and symptoms of TP	Hematochezia, petechiae, gingival bleeding, epistaxis	None	None	Epistaxis	Bleeding from tumor	None	None	None	None	None	None
Confirmation of ITP	Bone marrow biopsy	—	Peripheral smear	—	—	—	—	—	—	—	—
Treatment 1/highest PLT	MePRDL + IVIG/18	None required	None required	Prednisolone + IVIG/30	Prednisone/118	Prednisone/307	None required	None required	None required	None required	None required
Treatment 2/highest PLT	Rituximab + prednisone/364	—	—	None required	None required	^a^Prednisone/269	—	—	—	—	—

Entries with “—” indicate not applicable to patient

*Ipi* ipilimumab, *Nivo* nivolumab, *Pembro* pembrolizumab, *PR* partial response, *PD* progression of disease, *SD* stable disease, *N/A* not available, *TP* thrombocytopenia, *GI* gastrointestinal, *irAEs* immune-related adverse events, *WBC* white blood count, *HCT* hematocrit, *PLT* platelet, *MePRDL* methylprednisolone, *IVIG* intravenous immunoglobulin

^a^Patient relapsed after initial steroid treatment

Of the 11 patients, four required immunosuppressive treatment with corticosteroids, and two of those cases were refractory to steroids. A higher percentage of patients treated with ipilimumab (single agent or combined with nivolumab) required immunosuppressive treatment (75%, 3 of 4) compared to those treated with anti-PD-1 monotherapy (14%, 1 of 7). The majority of patients displayed no clinical signs or symptoms of thrombocytopenia and required no therapies with spontaneous resolution (Table 1). Our first case described in detail above had the most severe episode of thrombocytopenia with confirmed ITP by bone marrow biopsy.

## Conclusions

Thrombocytopenia, especially ITP, induced by immune checkpoint inhibitors appears to be a relatively uncommon irAE that is manageable with standard treatment algorithms. In our series, the majority of patients had mild thrombocytopenia that resolved spontaneously or responded to standard corticosteroid regimens. However, in two severe cases, steroids, IVIG, and rituximab were administered with ultimate recovery. In the first case, nivolumab monotherapy was resumed with excellent tolerance. On the other hand, the second patient relapsed with subsequent immune checkpoint inhibition.

Primary ITP is a disorder caused by the formation of autoantibodies targeting platelet antigens, leading to thrombocytopenia [15]. ITP is a diagnosis of exclusion and may be challenging given the lack of specific testing and a wide differential diagnosis. ITP is thought to occur after an inciting event activates or alters the immune system, such as an infection, hematopoietic malignancy, or pharmacologic immune checkpoint inhibition [16]. However, most cases are idiopathic in etiology. A majority of acute cases (50–90%) are responsive to standard corticosteroid and IVIG therapy, though a fraction of cases require second-line treatment, usually involving a combination of rituximab and a thrombopoietin agonist [17]. In mouse models, there is loss of peripheral self-tolerance through alteration of immune homeostasis and evidence of regulatory T cell (Treg) deficiency associated with ITP [18]. Comparison of bone marrow between patients with ITP and normal donors revealed that those with ITP have lower levels of Tregs and abnormal levels of Th1 and Th17 cells [16]. Recent work demonstrated that patients with chronic ITP exhibit lower levels of PD-1 expression in total peripheral blood samples, compared with healthy controls [19, 20]. A single case report showed that a patient who developed nivolumab-induced ITP had higher PD-1 expression on B cells [11].

Our experience suggests that thrombocytopenia, including ITP, may rarely complicate immune checkpoint inhibitor therapy but is usually mild and can resolve spontaneously or with standard treatment algorithms. The onset of ITP varies substantially, though a majority occurs within the first 12 weeks after initiation of checkpoint inhibition, consistent with other irAEs [21–23]. Although our observations on checkpoint inhibitor rechallenge after resolution of ITP are limited, our experience suggests that increased clinical vigilance should be used, especially with ipilimumab.

## Abbreviations

CTLA-4: Cytotoxic T-lymphocyte antigen 4
irAE: Immune-related adverse event
ITP: Immune thrombocytopenic purpura
IVIG: Intravenous immunoglobulin
PD-1: Programmed death 1
PD-L1: Programmed death ligand 1
PD-L2: Programmed death ligand 2
Treg: Regulatory T cell
